# Systematic Search and Evaluation of mobile Apps for Wound Care Available in French-Language in Canada

**DOI:** 10.1177/08445621241312394

**Published:** 2025-02-17

**Authors:** Julie Gagnon, Julie Chartrand, Sebastian Probst, Éric Maillet, Emily Reynolds, Valérie Chaplain, Heidi St-Jean, Raphaelle East, Michelle Lalonde

**Affiliations:** 1 School of Nursing, 70363 Faculty of Health Sciences, University of Ottawa, Ottawa, Ontario, Canada; 2 Département des sciences de la santé, 70348 Université du Québec à Rimouski, Rimouski, Québec, Canada; 3 Children's Hospital of Eastern Ontario Research Institute, Ottawa, Ontario, Canada; 4 HES-SO, University of Applied Sciences and Arts Western Switzerland, Geneva, Switzerland; 5 Faculty of Medicine, Nursing and Health Sciences, 2541 Monash University, Melbourne, VIC, Australia; 6 College of Medicine, Nursing and Health Sciences, 8799 University of Galway, Galway, Ireland; 7 Care Directorate, 27230 Geneva University Hospitals, Geneva, Switzerland; 8 School of Nursing, Faculty of Medicine and Health Sciences, 7321 University of Sherbrooke, Sherbrooke, Québec, Canada; 9 Monfort Hospital, Ottawa, Ontario, Canada; 10 89545 Edmundston Regional Hospital, Edmundston, New-Brunswick, Canada; 11 Faculté de médecine, 4440 Université Laval, Québec, Canada; 12 Institut du Savoir Montfort, 153164 Montfort Hospital, Ottawa, Ontario, Canada

**Keywords:** Mobile applications, mobile health (mHealth), wound care, review, user mobile app rating scale, Canada

## Abstract

**Background:**

Wounds are a significant national health concern, impacting individuals, healthcare systems, and the environment. Despite efforts by organizations to promote evidence-based practices, gaps persist between theory and nurse practice in wound care. Mobile apps show promises in enhancing wound care delivery, but their rapid evolution, including adaptations into different languages such as French, raises concerns about reliability and regulation. Evaluating these apps is crucial for ensuring patient safety and effective wound management.

**Purpose:**

To review and assess mobile wound care apps available in French for healthcare providers in Canada.

**Methods:**

A systematic search was conducted across the literature and the two main Canadian online app stores (App Store and Google Play). The included mobile apps underwent quality evaluation using the user version of the Mobile Application Rating Scale (uMARS).

**Results:**

The initial search retrieved 1,550 apps, of which 260 were screened and 5 included. Included apps were from France and were available on both stores. These apps varied in features, including wound dressing directory (*n* = 3), best practices reminders (*n* = 2), photography management and digital wound tracking (*n* = 1), and total body surface area calculator (*n* = 1). Evaluation using uMARS indicated total averages range from 3.52/5 to 4.10/5. The results offer scant insight into the design and evaluation of the apps included.

**Conclusions:**

The study highlights the need for development and validation of a French wound care app tailored to Canadian healthcare contexts and best practice recommendations, emphasizing collaboration among nurses and stakeholders in technology enhancement for the benefit of Canadians’ health.

## Background and purpose

Wounds represent a major public health issue with significant impacts on individuals, the environment, and healthcare systems (Olsson et al., 2019; Reeves & Chaplain, 2023; Sen, 2023). Accurately estimating wound prevalence is challenging due to differences in study designs and measurement methods (Martinengo et al., 2019). However, chronic wounds, such as venous ulcers and pressure injuries, are estimated to affect 2.21 per 1000 people globally (Martinengo et al., 2019). The prevalence of chronic wounds is rising due to an aging population, sedentary lifestyles, and increasing rates of obesity and chronic diseases like diabetes (Martinengo et al., 2019; Sen, 2023). Treating chronic wounds is costly, burdens healthcare systems, and significantly reduces productivity (Guest et al., 2020; Nussbaum et al., 2018; Olsson et al., 2019). Beyond financial and environmental consequences, wounds increase mortality and negatively impact quality of life due to prolonged hospitalizations, pain, anxiety, social isolation, and amputations (Avsar et al., 2021; Olsson et al., 2019; Ratliff & Rovnyak, 2021; Roussou et al., 2023; Sen, 2023). According to data obtained from the Canadian Institute for Health Information's (CIHI) Discharge Abstract Database on all hospital discharges associated with lower limb amputation from April 2006 to March 2012, 14 Canadians were amputated daily due to diabetes-related complications (Imam et al., 2017), a number that has risen over the past decade (Mohamad et al., 2019).

In Canada, limited studies and lack of accessibility to public system data hinder our understanding of the current wound care situation (Blanchette & Kuhnke, 2021; Houghton, 2021). A 2011–2012 study conducted by the CIHI, indicates wound prevalence of 28.2% in complex continuing care^1^, 9.6% in long-term care, 7.3% in-home care, and 3.7% for acute inpatients. However, these results likely underreport cases. Also, it is important to note that these numbers exclude Quebec province due to data pooling challenges (CIHI, 2013; Denny et al., 2014). There is no recent data on the prevalence or incidence of wounds in Quebec province or francophone facilities. It is generally accepted that provincial data is similar. In this regard, the recent Patient Cost Estimator from CIHI provided data for Quebec based on estimates obtained from other provinces. This highlights the importance of creating digital tools to better understand the wound situation in Quebec, but also across Canada.

CIHI developed a tool to estimate the average cost per patient for services provided to hospitalized patients in short-term care in Canada, including length of stay (CIHI, 2023). For chronic ulcers, such as pressure injuries or lower limb ulcers, the estimated average cost per patient is $13,761, with 10–12 days of hospitalization for adult patients. For diabetic foot ulcers, the average cost is $12,100, with 8–10 days of hospitalization for adult patients. These estimates cover hospital expenses but exclude physician fees (CIHI, 2023).

Wounds affect all patient populations and remain a common challenge across all healthcare settings. Nurses play a critical role in wound healing and care. The complexity of clinical cases and the rapid evolution of treatments in a very broad nursing profession context make best practices dependent on current knowledge, clinical competence, and contextual realities (Orsted et al., 2018; Stacey, 2016). Evidence-based nursing care can help prevent or mitigate the deleterious effects of wounds, improve quality of life, and reduce costs (Kielo et al., 2019; Pham et al., 2012). This is the cornerstone of national organizations like Wounds Canada, a charitable organization dedicated to advancing wound prevention and management for all people in Canada, Nurses Specialized in Wounds, Ostomy, and Continence Canada, and the Institut National d’Excellence en Santé et Services Sociaux, which publish best practice recommendations, often in online PDFs. However, these tools require Internet access (not always available in community care), or involve printing costs, the transportation of bulky documents and the availability of outdated guidelines (Patel et al., 2019). Despite these resources and continuing education, a gap remains between theory and practice in wound care.

Notwithstanding the emphasis on evidence-based practices in wound care, many nurses still rely on their knowledge, experiences, and exchanges with colleagues (Gagnon, 2020; Gillespie et al., 2014; Hughes, 2016). This can perpetuate outdated methods and ritual practices. Additionally, the selection of dressings and local wound care often changes randomly across settings (Gray et al., 2018; Guest et al., 2020), leading to confusion and misalignment with the prescribed care plan (Gray et al., 2019).

Mobile apps have the potential to mitigate or overcome such barriers by enhancing wound care and support evidence-based practice (Beeckman et al., 2013; Kulikov et al., 2019; Moore et al., 2015; Patel et al., 2019; Shamloul et al., 2019; Zhang et al., 2021). Mobile Health (mHealth), defined as “medical and public health practice supported by mobile devices” (World Health Organization, 2011, p. 6), offers clear benefits. For patients, it enables more accurate assessments, early treatments, complication prevention, and better clinical outcomes. For nurses, it facilitates clinical decision-making, boosts confidence and reduces isolation in providing care across Canada's vast territory by enabling remote consultations with wound care specialists. For the healthcare system, it reduces costs and improves care delivery in remote areas where expertise is less accessible (Kulikov et al., 2019; Moore et al., 2015; Patel et al., 2019).

A 2023 national survey by Canada Health Infoway (*n* = 1,907) (2024), found that nine out of ten nurses (91%) providing direct patient care surveyed use electronic record or clinical information systems, and 50% use electronic clinical decision support tools. The latest available data show that over 70,000 mHealth apps are publicly available in the medical category on Google Play and the App Store globally (Statista, 2024), creating a challenge for nurses and healthcare organizations to choose the right app for their needs, and one that offers quality, reliable content from a legitimate, credible authority. The number of mobile health apps available on these stores fluctuates continually. Despite the potential of mobile apps to improve nursing practices, their rapid evolution raises concerns and potential risks, such as substituting clinical judgment, duplicating actions, or documentation, or using unreliable applications (Canadian Nurses Protective Society, 2013). Although the number of mobile apps is growing, evidence on their development and impact remains limited (Marcolino et al., 2018). Research is not keeping pace with the exponential growth of mobile apps available to nurses (Jogova et al., 2019).

Additionally, the lack of regulation makes it challenging to identify reliable mobile apps among the many digital tools available. The World Health Organization (World Health Organization, 2019) recognizes this issue and emphasizes the importance of validating the information used in mobile app algorithms. Given the central role of nurses in wound care, their involvement in assessing and evaluating available mobile apps in this field is crucial. Previous studies have highlighted concerns about the unsupervised development and evaluation of mobile apps, which can jeopardize patient safety, be influenced by commercial biases, and pose privacy risks (Dege et al., 2024; Koepp et al., 2020; Patel & Thind, 2020; Wurzer et al., 2015).

With 327 million speakers, French is the fifth most spoken language in the world (Observatoire démographique et statistique de l’espace francophone, 2024). Canada has two official languages: English and French, the latter representing 27.51% of the entire population (Observatoire démographique et statistique de l’espace francophone, 2024). Although both languages are spoken across the country, French remains the majority language in the province of Quebec, which, according to the same source, is home to 73,37% of French-speaking Canadians. Nearly one in five Canadians can converse in both English and French (Statistics Canada, 2023). Despite this, no reviews or evaluations of mobile wound care applications in English or French have been identified in Canada.

## Aims

Wanting to assess the current situation and develop a mobile application initially dedicated to healthcare professionals in Quebec, the aim was to review and assess French-language mobile wound care apps retrieved in Google and Apple App Store for healthcare providers in Canada. The objectives were to:

1. Conduct a literature review and survey in the Canadian Google and Apple app stores to identify relevant wound care apps in French;
2. Assess the quality features of these identified apps.

## Methods and procedures

### Study design and eligibility criteria

A pragmatic approach (Dewey, 1931) and a hybrid model (Lau et al., 2021) were used to conduct this research. John Dewey's pragmatic approach (1931) is based on the idea that knowledge and truth emerge through experience and practice. This is why particular importance was placed on the usefulness and effectiveness of apps in clinical practice, and their adaptation to the context of wound care in Canada. Furthermore, Dewey (1931) emphasizes the importance of collaboration in solving collective problems. In this research, this is reflected in the collaboration of a team involving clinicians and researchers. The hybrid model proposed by Lau et al. (2021) is an innovative approach that combines a traditional systematic literature review with a systematic search of mobile app download platforms. So, the research was conducted in three steps: 1) literature review, 2) systematic search, and 3) quality assessment of mobile app platforms. The criteria for the targeted apps are presented in Table 1.

**Table 1.** Eligibility criteria for the targeted apps.

Included	Excluded
- Apps used in clinical practice for wound care (e.g., teleconsultation, assessment, dressing selection, treatment planning, education, etc.) - Apps available in Canadian online stores (App Store or Google Play) - Apps available in French - Apps intended for healthcare professionals of all ages and education levels - Apps for mobile phones or tablets - Apps for any care settings (hospitals, community settings, primary care facilities, and all other care settings where wounds were privately or publicly managed) - No restrictions on the country of origin - No restrictions on developers (companies, government, research, other) - No restrictions on cost	- Apps intended for patients (self-care or other) - Apps not exclusively focused on wound care (e.g., general clinical decision support tools) - Apps for general telemedicine and teleconsultation, or general communication platforms (e.g., FaceTime, Skype) - Apps for long-term training - Apps for viewing standalone PDFs, full volumes, online textbooks, or journals - Apps for professional exam preparation - Apps requiring external electronic information systems or other external devices - Apps for general skin problems or dermatology only - Apps intended for non-human care (e.g., veterinary) - General first aid apps - Gaming apps - Apps developed exclusively for laptops or desktop computers - One-off applications for congresses or conferences - Non-functional applications

### Step 1 – Traditional literature search

The literature was systematically searched to identify eligible mobile apps. The search strategy, based on previous research (Gagnon et al., 2024), utilized keywords derived from our knowledge and an exploratory search in the titles, abstracts, thesaurus (MeSH), or subheadings of MEDLINE (via Ovid), Cumulative Index to Nursing and Allied Health Literature (CINAHL via EBSCO), and Embase. Supplementary File 1 presents the MEDLINE strategy. The final strategy was translated into PubMed, MEDLINE (via Ovid), Embase, CINAHL (via EBSCO), Web of Science, ScienceDirect and Scopus, LiSSa (Littérature Scientifique en Santé), Cochrane Library, Institute of Electrical and Electronics Engineers (IEEE) Xplore digital library, and Érudit, to identify mobile apps available in French in Canada. All original full-text publications presenting, developing, or evaluating wound care mobile apps for healthcare providers were considered for the search of eligible apps. This encompassed various study types and sources, including clinical research, quantitative and qualitative studies, literature reviews, protocols, conference abstracts, theses, editorials, government reports, and guidelines. Grey literature was sourced from the Nursing and Allied Health Premium (ProQuest), ProQuest Dissertations and Theses, Global Index Medicus (WHO), OpenGrey (1980–2020), Grey Literature Report (1999–2016), National Institute for Health and Care Excellence (NICE), Wounds Canada, European Wound Management Association, American Professional Wound Care Association and WorldWideScience. Study registers were also screened via Prospero, ClinicalTrial.gov, and Cochrane Central Register of Controlled Trials. Thorough research encompassed comprehensive studies ensued from protocols, along with potential articles extrapolated from conference abstracts. Publications in French or English were included. Articles solely presenting app coding were excluded. No time limit was imposed on search results due to the evolving mHealth concept. Searches were conducted between April and August 2023, with an update in March 2024. Manual searches of citation lists were conducted for additional articles.

All sources of evidence were managed using Covidence (Veritas Health Innovation, Melbourne, Australia). Duplicates were removed and eligibility criteria were specified. A pilot test was conducted before beginning the source selection process. The first 10 sources of evidence were selected, and the reviewers screened them using the eligibility criteria and met to discuss any discrepancies. The pilot test achieved a 92% interrater agreement. No modifications were made to the eligibility criteria, and the initial blind screening of all titles and abstracts was conducted by two independent reviewers. Full texts of potentially relevant publications were obtained and integrated into Covidence. Publications failing to meet the eligibility criteria were excluded, and the reasons for exclusions were recorded in an Excel spreadsheet (Microsoft 365). Any disagreements were resolved through consensus, eliminating the need for a third reviewer. Data on the identified mobile apps were extracted using the AI research assistant Elicit (version March 2024), verified independently by two research team members, and entered into an Excel data extraction table. Descriptive data included app name, developer and affiliation, country, cost, language, platform, mobile device, purpose, users, version, date of last update, conception process (e.g., theoretical background, expert involvement), Internet access requirements, technical features (e.g., sharing capabilities, community presence), security and confidentiality aspects (e.g., login requirements, detailed privacy policy). Missing information was obtained by contacting developers.

### Step 2 – Systematic search of mobile app platforms

A systematic search of online app stores was essential to obtain a comprehensive overview of available apps. In Canada, 62.06% of smartphone users utilize Apple devices, while 37.41% use Android devices (Statcounter GlobalStats, 2024). Therefore, the search focused extensively and iteratively on the two primary mobile platforms and their respective online app stores: the Google Play Store for Android devices and the Apple App Store for iOS devices. Search terms from the first step were entered in both English and French search engines of each online app store to systematically identify eligible apps. Due to limitations in logical operations and truncation in online app stores, search terms had to be entered individually. The “Medical” and “Health and Wellness” store categories were particularly scrutinized. Clearing cookies before each search minimized unintentional bias. Additionally, apps were searched through various websites, including Health Canada, the Mobile Centre of the Government of Canada, 137 recognized schools of nursing libraries according to the Canadian Association of Schools of Nursing (2022), provincial health ministries, iMedicalApps, MédecinGeek, MobileAction, Facebook, and LinkedIn.

Two authors independently assessed all apps against the eligibility criteria. An initial screening was conducted between August and November 2023, followed by a renewed search in March 2024 using the latest app versions. A comprehensive Excel spreadsheet categorized included and excluded apps, with relevant data extracted from the apps, app stores, literature, or official websites. Duplicate apps within the same store were removed manually, and duplicates across both app stores were carefully examined to identify distinctive features and consolidate data. Each stage, such as initial screening, full-app review, and data extraction, was completed by two separate reviewers. They resolved ambiguities among each other until they reached consensus.

### Step 3 – Quality assessment of the included apps

The mobile apps identified in steps 1 and 2 were combined and downloaded onto a mobile device full-app review to determine eligibility. The cost of purchasing the only paid application was covered by research funds. Apps that did not meet the eligibility criteria were excluded, with reasons recorded in the Excel spreadsheet.

The included mobile apps underwent quality assessment employing the uMARS (Stoyanov et al., 2016). This scale is a simpler, end-user version of the Mobile Application Rating Scale (MARS), which is a multidimensional, reliable, and flexible tool commonly used by researchers and healthcare professionals to effectively assess the quality of mHealth applications (Azad Khaneghah et al., 2020; Stoyanov et al., 2015; Terhorst et al., 2020). It demonstrates excellent internal consistency (Cronbach alpha = 0.90) with high individual alphas across all subscales scores and demonstrates good test-retest reliability (Stoyanov et al., 2016). The uMARS has widespread utilization in various research endeavours (Hajesmaeel-Gohari et al., 2022; Lambrecht et al., 2021; Villalobos et al., 2022), including a study related to wound care apps (Dege et al., 2024).

This scale consists of 20 items rated on a five-point scale (ranging from “1.Inadequate” to “5.Excellent”), organized into four objective evaluation sections: engagement, functionality, aesthetics, and information quality. The overall app quality is determined by calculating the average of these four sections. An additional subjective evaluation section provides insight into personal appreciation. The items within last section are also rated on a five-point scale, with the average score calculated to obtain the “subjective quality rating of the application.” This final section is independent of the others objective sections, focusing on questions related to personal appreciation. The “Perceived impact” section was not accounted for in this study as the apps were not linked to a specific health behaviour target.

**Figure 1.**
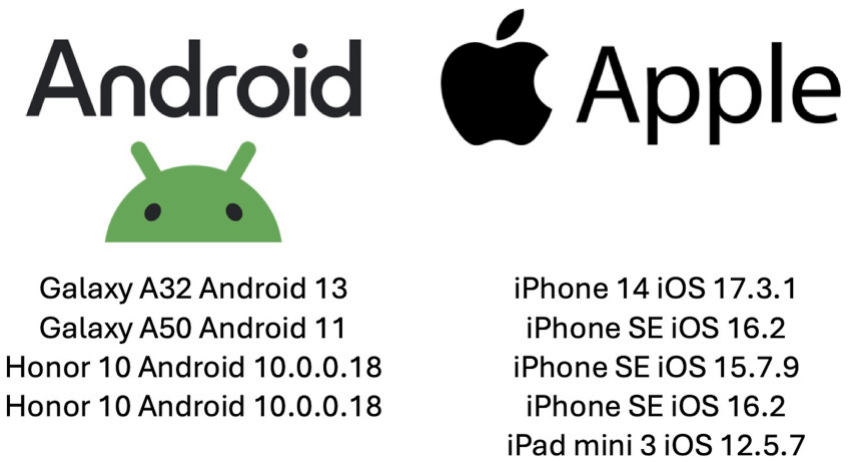
Devices used to evaluate the apps.

Three authors nurses specialized in wound care, ostomy, and continence from French-speaking regions of Canada (New Brunswick, Ontario, and Quebec) evaluated each application. The evaluation was conducted using various smartphone devices and a tablet (Figure 1).

All evaluations were completed between April 1 and April 14, 2024, with each app independently tested before assessing uMARS criteria. After evaluating all the apps, the average results from the three raters were calculated to obtain category scores and an overall score for each app. Inter-rater reliability among the three raters was assessed to ensure quality (Belur et al., 2018). Absolute agreement between raters was determined using intraclass correlation within a two-way mixed-effects model. Results, presented with a 95% confidence interval, were categorized as follows: poor for values below 0.5, moderate between 0.5 and 0.75, good, and excellent for values exceeding 0.90 (Koo & Li, 2016).

### Data analysis and synthesis

Results from the uMARS evaluations were calculated for each included application and a descriptive statistical analysis was conducted using Excel software. The average of the items in each section was calculated to obtain a “section average score” on a five-point scale, excluding questions rated as “N/A” from the mean score calculation of the last section. The final score for each application was determined based on the overall mean rating (Stoyanov et al., 2016). Descriptive findings from the systematic search and uMARS scales were summarized and integrated into a summary table.

Approval from research ethics boards was not required as data were obtained from publicly available platforms.

## Results

### App screening

**Figure 2.**
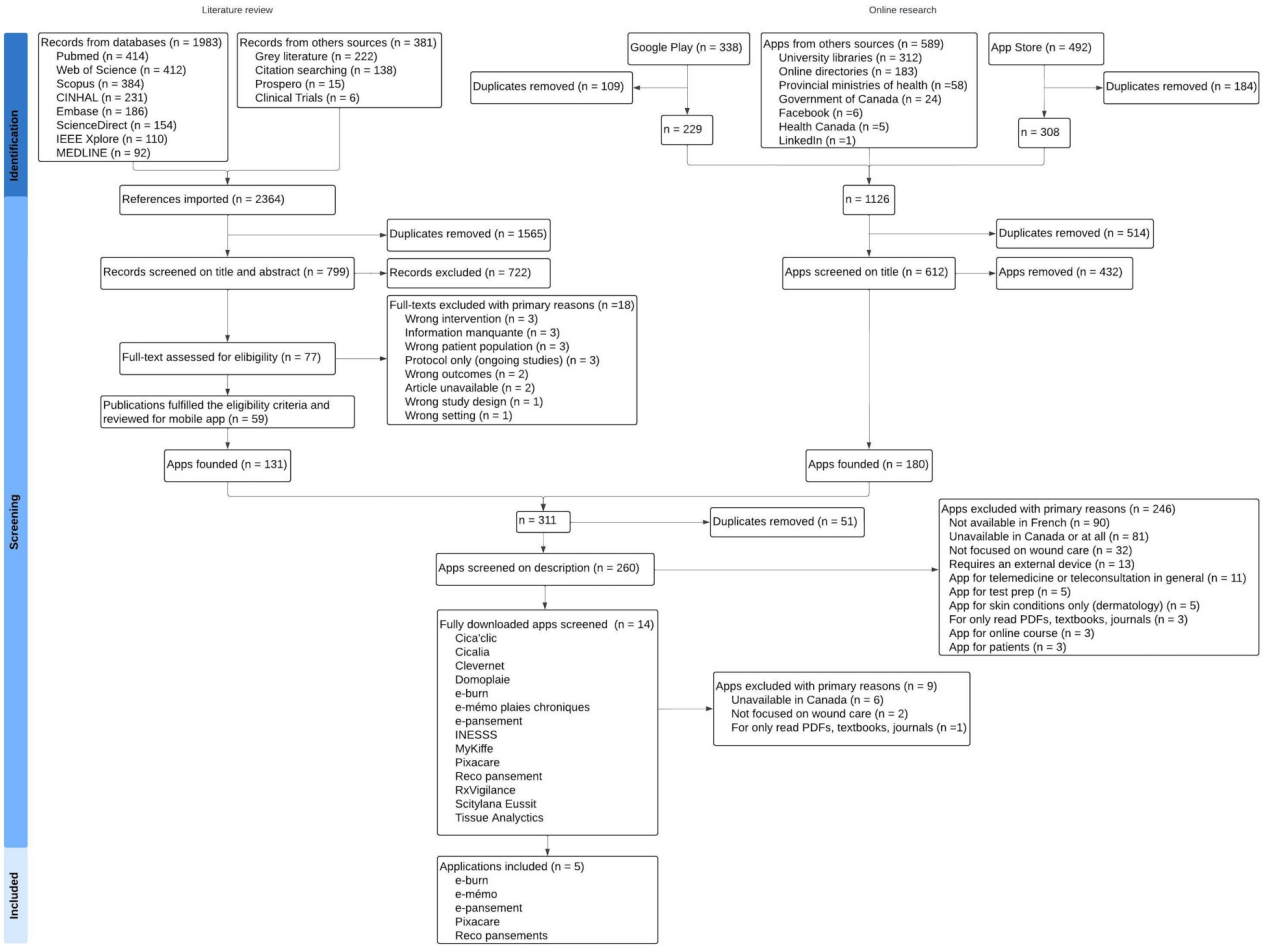
Study flowchart.

A total of 1,550 apps were retrieved, including 131 from the literature (8.5%). After removing duplicates and irrelevant apps, 260 apps from the initial 1,550 (16.8%) remained. Subsequently, 246 apps of these 260 (94.6%) were excluded based on available online app store summaries. Fourteen applications were subsequently downloaded. After contacting developers, the applications “Cica’clic”, “Scitylana Eussit”, and “Tissue Analytics” were excluded because they are not available for Canadian healthcare providers (a license number or login from outside Canada is required). The developers of the “RxVigilance” app were also contacted and confirmed that the wound care module is not available in the mobile app of their specialized medication software. After downloading and screening the apps, an additional nine were excluded. Figure 2 illustrates the flowchart depicting the steps in applying the eligibility criteria. The list of all excluded applications and the primary reasons for their exclusion, such as unavailability in French-language (*n* = 90, 35.3%) or the app being unavailable in Canadian app stores (*n* = 87, 34.1%), are detailed in Supplementary File 2.

Finally, five apps were included in the review, namely “e-burn”, “e-mémo plaies chroniques”, “e-pansement”, “Pixacare”, and “Reco Pansement AP-HP”. All included apps were found using both the App Store and Google Play. Four of them were also referenced in the literature: the “e-burn” app was discussed in a letter to the editor of a peer-reviewed journal (Fontaine et al., 2018), the “e-pansement” app was described in an article from a professional review (Klein, 2016), as well as in a comparative study of mobile apps conducted in a thesis (Fontaine, 2020). The thesis also mentioned the “e-mémo plaies chroniques” and “Reco Pansement AP-HP” apps.

### App features

Table 2 summarizes the features of the included apps, all from France. The sources of these apps vary: two from government agencies, two from companies, and one from a nonprofit organization. Four types of apps were identified: wound dressing directory (*n* = 3), best practices reminders (*n* = 2), photography management and digital wound tracking (*n* = 1), and Total Body Surface Area (TBSA) calculator (*n* = 1). The “e-mémo plaies chroniques”, “e-pansement”, and “Reco Pansement AP-HP” apps provide wound treatment directories and reference materials. These resources facilitate direct access to essential information for patient evaluation and management, including a directory of wound dressing options. However, these apps do not feature specific algorithms. They do not support patient record creation or integration with electronic health records. In contrast, the “Pixacare” app empowers users to generate digital patient records, synchronize data with existing records, and track and share photos, facilitating telemonitoring. Nevertheless, it also lacks specific treatment or management algorithms. On a different note, the “e-burn” app provides a fixed algorithm for calculating the burned TBSA, using a draw representation on a template (Lund and Browder chart). It also calculates the required fluid administration over the initial 24-h post-injury period using the Parkland formula.

**Table 2.** Included apps (in alphabetical order).

Name and release date	Platform, device, and minimal version of operating system	Developer (affiliation) and country	Price	Downloads	Description	Users	Conception process or validation	Last update	Internet requirement	Privacy policy available
e-burn Premium (Fontaine et al., 2018) May 21, 2021 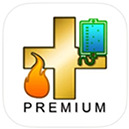	App Store iPhone, iPad, iOS 11.0 Google Play Android 4.4	BreakFirst (NGO^a^) France	$2.99 +tx	Google Play: 500+	Accurate TBSA measurement. Recommendations of the French Burns Society included. To guide initial fluid management (adult or child).	Healthcare professionals	Program developed in partnership with Intensive Care Burn Unit Team at the Hôpital Saint Joseph Saint Luc (Lyon, France).	Oct. 2023	No	Yes (on Google Play only). Allows results to be shared.
e-mémo plaies chroniques Feb. 15, 2016 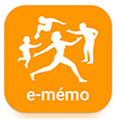	App Store iPhone, iPad iOS 6.0 Google Play Android 4.0	L’assurance Maladie (Government) France	Free	Google Play: 10,000+	To guide initial assessment and follow-up of wounds. Addresses elements of prevention. Access to exemplary recommendations in the form of tables, decision trees, images, diagrams, and photos.	Physicians and nurses	Content validated by the Société Française et Francophone des Plaies et Cicatrisations and the Haute Autorité de Santé.	Sept. 2016	No	No
e-pansement (Klein, 2016) Nov. 29, 2012 Website 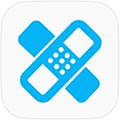	App Store iPhone, iPad iOS 10.0 Google Play Android 4.2	Elevate SAS (Company) France	Free	Google Play: 50,000+	Help in identifying and categorizing wounds, guide to identifying the different wound stages, and dressing selection. Full description of dressings available on the French market. Recommendations and best practice guides. News feed. Preregistration for approved training courses.	Healthcare professionals	Created by healthcare professionals. Based on Haute Autorité de Santé and European Wound Management Association best practices. Total independence.	App Store: Nov. 15, 2021 Google Play: Feb. 5, 2024	Most functions are available without Internet connection.	Yes Possible to share on social networks.
Pixacare 2020 Website 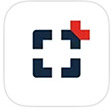	App Store iPhone iOS 13.6 Google Play Android 7.0	Pixacare (Company) France	Free	Google Play: 1000+	Wound photo management, remote monitoring, and follow-up. View all photos taken by the team. Send reminders. Share photos and exchanges between users in a secure messaging system.	Healthcare professionals	N/A	Apple: Jan. 15, 2024 Google: Oct. 25, 2023	Can take photos without a network connection but the Internet is required for all other features.	Yes Requires login and password
Reco Pansements AP-HP July 31, 2019 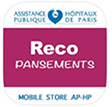	App Store iPhone, iPad iOS 9.0 Google Play Android 4.1	Assistance Publique - Hôpitaux de Paris (AP-HP) (Government) France	Free	Google Play: 1000+	Recommendations for effective wound dressing selection to promote healing.	Healthcare professionals	Based on the clinical practices and advice of a multidisciplinary expert group from the AP-HP. Credible sources of information identified.	App Store: 2023 Google Play: Aug. 19, 2022	No	Yes

^a^ Non-governmental organization.

All four apps intended to support clinical decisions indicated clinician involvement in app development, assessment by experts in their related field, or affiliation with credible European organizations. Furthermore, they described sources of information, either in a bibliography or integrated within the app. Only the “Pixacare” app requires a login and password, securing encrypted data on servers based in France and Frankfurt, certified for health data hosting in both the United States and France. While standard privacy and data security policies are available for the other apps, “e-mémo plaies chroniques” lacks specific details in this regard.

### App quality assessment

Table 3 presents uMARS scores of the five included applications. Overall, the applications scored above the acceptable minimum score of 3.0 in all five categories. The total averages for each application range from 3.52 to 4.10, indicating overall quality falling between “acceptable” and “good” (Stoyanov et al., 2015). The average across all applications is 3.84 ± 0.22. Notably, the “e-pansement” app received the highest overall quality score with 4.10, appreciated for its quick access to clinical guidelines and updates, while the “Reco Pansements AP-HP” app received the lowest score with 3.52.

**Table 3.** Scores of included applications.

	App quality mean score (SD)^a^					Subjective mean score
	Engagement	Functionality	Aesthetics	Information	Overall mean app quality total score	
e-burn Premium	3.66 (0.72)	4.08 (0.29)	4.22 (0.67)	3.92 (0.51)	3.97 (0.24)	3.08 (0.51)
e-mémo plaies chroniques	2.93 (1.03)	4.33 (0.49)	3.67 (0.71)	4.33 (0.49)	3.82 (0.67)	2.67 (0.49)
e-pansement	3.47 (1.13)	4.67 (0.49)	3.78 (0.67)	4.50 (0.52)	4.10 (0.57)	3.17 (0.58)
Pixacare	3.60 (0.63)	4.50 (0.52)	4.00 (0.00)	3.20 (0.43)	3.83 (0.56)	3.58 (0.29)
Reco Pansements AP-HP	2.40 (1.06)	4.33 (0.49)	3.67 (0.5)	3.67 (0.65)	3.52 (0.81)	2.08 (0.90)

^a^ Each score is based on uMARS five-point scale from 1-Inadequate to 5-Excellent.

**Figure 3.**
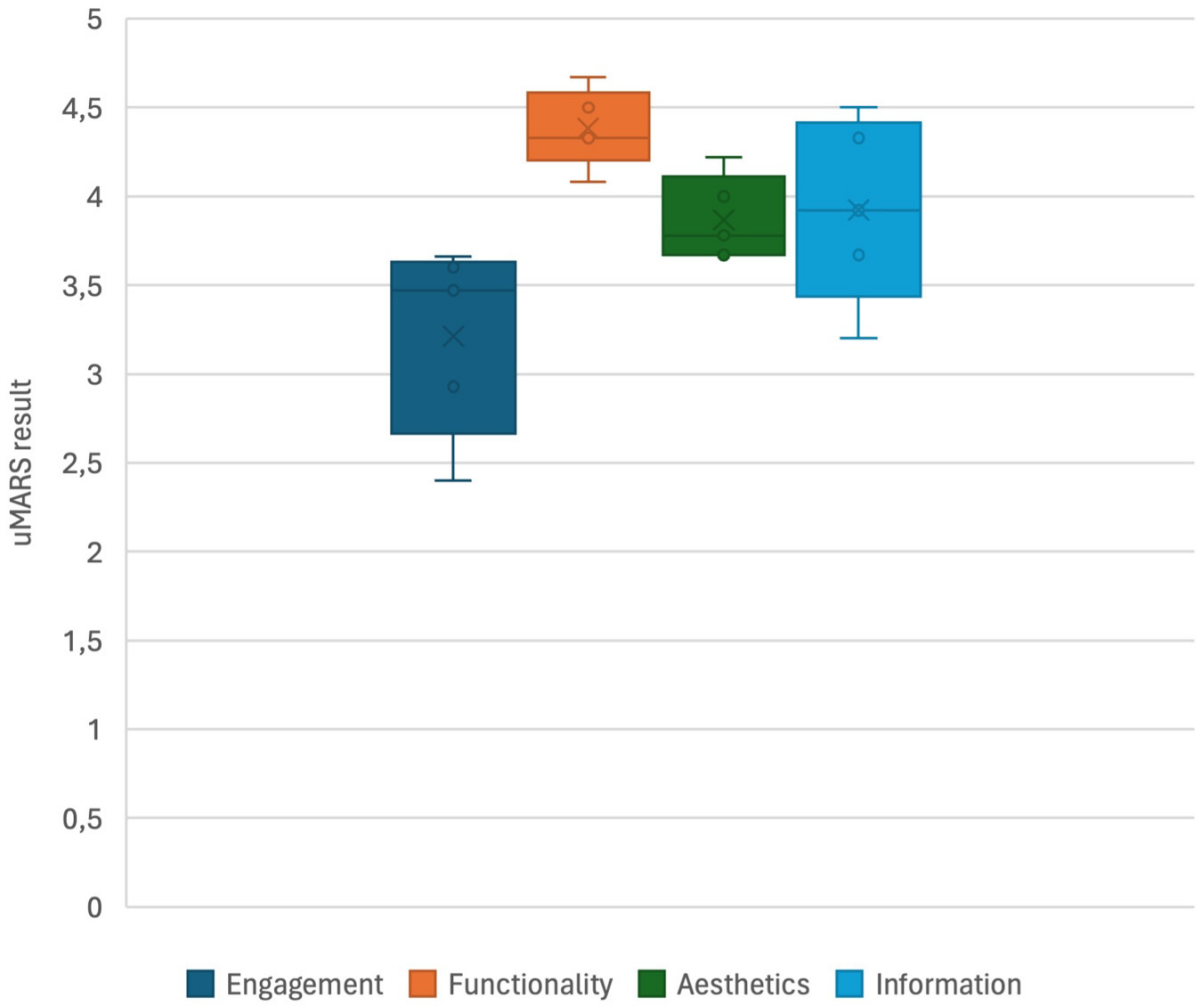
Graphical representation depicting the overall rating distribution of uMARS scores, showcasing means, medians, interquartile ranges, and ranges across five mobile apps (*n* = 5).

Subjective evaluations conducted by the authors using uMARS averaged 2.92 ± 0.57. Among the apps, “Pixacare” received the highest average subjective score (3.58), while “Reco Pansements AP-HP” received the lowest (2.08). Inter-rater reliability of all apps was deemed good (ICC 0.90, 95% CI 0.86–0.93). Detailed analysis of average subscale scores for all applications revealed functionality rated highest, while engagement rated lowest (Figure 3).

## Discussion

This research involved three steps, a literature search and examination of mobile wound care apps available in French-language on Canadian App Store and Google Play platforms. Five applications with various purposes were included and evaluated. Due to stringent eligibility criteria, particularly language requirements, only a small subset of apps could be analyzed in this study, which extended over a considerable period. Comparing the number of included applications with previous studies proves to be challenging due to their methodological variations. Previous research reports have shown a wide range, varying from 3 to 66 applications (Dege et al., 2024; Fontaine, 2020; Koepp et al., 2020; Schnalzer et al., 2022; Shamloul et al., 2019; Wurzer et al., 2015). A key finding was the absence of Canadian-originated apps among those included. All included applications are from France, and some components cannot be directly transposed to the Canadian healthcare system, such as the availability and approval of pharmaceuticals and dressings. Even company-developed apps were unavailable to Canadian healthcare professionals (such as “Cica’clic” and “Mépi-Coach” apps). Despite the quality of the selected apps, some discrepancies in terminology and availability were observed. This absence underscores the untapped potential for innovative digital solutions to address nurses’ clinical needs and ensure compatibility with current and future electronic health records within the large Canadian healthcare system. Future initiatives should focus on developing and validating apps that support contextually relevant clinical decisions within the Canadian healthcare system, guided by national best practices.

The most common reason for exclusion was the lack of available French-language apps. Considering the proportion of bilingual Canadians, evaluating the quality of these excluded apps remains essential and warrants further investigation. Canadian-developed apps like “WounDS” and “Swift”, which are currently only available in English, deserve particular attention. “Health Espresso” is also an app to keep a close eye on because it is created in partnership with Wounds Canada to digitize a patient's journey and connect members of allied health teams at the right time to decrease acute and hard-to-heal wounds, reduce hospitalizations and improve patient outcomes (Wounds Canada & Health Espresso, 2022). Despite the limited number of apps in this study, a significantly larger quantity remains accessible in Canadian online app stores. This has a major impact on nurses’ search for the “right” application for clinical decisions, while having to ensure they understand the specialized terms. This issue of app overload has persisted for over a decade and has only gotten worse over time (van Velsen et al., 2013). Consistently, the conclusion remains unchanged: validation efforts are struggling to keep pace with the rapid production of mobile apps.

The included applications underwent evaluation using uMARS, a multidimensional questionnaire for assessing app quality among end users (Stoyanov et al., 2016). The inter-rater agreement indicated good reliability in the assessment process (Koo & Li, 2016). The average score for each application fell between “acceptable” and “good” (Stoyanov et al., 2015). This outcome is consistent with a study by Dege et al. (2024), which systematically identified and assessed publicly available apps for patients with chronic wounds on the German Google Play Store and App Store. In their study, the three retained mobile apps, evaluated by 10 physicians, obtained scores ranging from 2.64 ± 0.65 to 3.88 ± 0.65. Significant differences exist among the included applications across evaluation categories. Only one app (“e-pansement”) achieved an average score exceeding four points, qualifying as “good.” The lowest averages were observed in the “Engagement” category, suggesting that developers may not adequately focus on user interest and interactivity. Both objective and subjective evaluations revealed that the “Reco Pansements AP-HP” app had the lowest scores, particularly in “Engagement” (2.08). This could be attributed to the app's heavy reliance on textual content and lack of visual elements. To confirm this hypothesis, it would have been useful to further question nurses specialized in wound care regarding the included mobile applications. Although the uMARS allows for a quantitative subjective evaluation, future research should include qualitative data collection to better understand and contextualize the results.

Most of the apps found in literature originate from systematic or search reviews. The results provide very little information regarding the design and evaluation of the selected apps. The development and evaluation of mobile wound care applications often lack rigorous scientific processes, potentially exposing users to invalidated content (Koepp et al., 2020; Moore et al., 2015). Few health-related mobile apps undergo thorough validation based on high-level evidence, raising safety concerns (Llorens-Vernet & Miró, 2020). In this study, most apps were developed in collaboration with specialized teams or validated by experts, or recognized bodies, like the Société Française et Francophone des Plaies et Cicatrisations. Nevertheless, the results underscore the necessity for comprehensive evaluations of their clinical efficacy and economic impact. This reinforces the point made by Lau et al. (2021) that less than 1% of mHealth applications have been the subject of scientific publications describing their effectiveness.

While apps providing clinical recommendations cite reliable references, a concern arises regarding update frequency. For instance, the “e-mémo plaies chroniques” app last updated in fall 2016, rendering it outdated despite remaining available on app stores. This underscores the challenge of maintaining and updating apps throughout their lifecycle (Mannino et al., 2023). Neglected apps will inevitably require complete code rewrites every one to three years, especially considering the rapid evolution of technologies and wound treatments. Regular clinical and technological updates are vital for healthcare professional-targeted apps.

Results indicate that four out of five applications have a data privacy policy available. Only the “Pixacare” app, which collects sensitive data and photos, requires a login and password. Consequently, anyone can download and use the other applications without restrictions. This global issue is being addressed by various governmental entities, such as the National Health Service in England, the Haute Autorité de Santé in France, and the Food and Drug Administration in the United States, which are developing national regulations to better govern mHealth. Health Canada has published draft guidelines on the regulation of software as medical devices (Government of Canada, 2019). However, mobile apps can still be made available to the public without any interaction with Health Canada (Jogova et al., 2019; Zawati & Lang, 2019). These results highlight the importance of harmonizing regulatory instruments worldwide for the safe adoption of mHealth solutions. Data security is crucial in the adoption of mobile technologies in healthcare. Privacy, data sharing disclosure, traceability, and transparency are essential factors not always detailed in policies available on online app stores.

When creating or using mHealth technologies in nursing care, it is crucial to understand how these technologies integrate into existing legislative and organizational frameworks. The apps retrieved and assessed in this systematic search serve as incentives to develop a mobile app tailored to French-speaking nurses and adapted to the Canadian healthcare system. To achieve this, the participation of nurses is essential through intercommunication between clinicians, researchers, and developers (Canadian Nurses Association & Canadian Nursing Informatics Association, 2017). Above all, an application's ability to impact health outcomes depends not only on the intervention's safety and effectiveness but also on the security and quality standards of the environment in which it is developed, distributed, promoted, and used (Grundy, 2022). Collaboration and cohesion among different stakeholders are essential, and nursing leadership is pivotal in improving wound care through communication and information technologies.

## Strengths and limitations

This study marks the first comprehensive and systematic effort to examine and rate currently smartphone apps available in French that are specifically designed for healthcare providers involved in wound care in Canada. Its strengths lie in its rigorous methodology, extensive screening of applications, and high inter-rater reliability. The main limitation of this research is the short timeframe of its results. The mHealth app market is constantly evolving, and the findings may only represent a snapshot of mHealth applications available in March 2024. Continuous monitoring is necessary to provide ongoing insights for nurses using such applications. App availability was based on the geolocation of the App Store and Google Play, which was set to Canada and not other countries. Exploring other French-speaking countries would be an interesting research avenue to increase the number of French-language apps available for evaluation and explore other technological initiatives. Moreover, the exclusion of apps unavailable on the App Store and Google Play may limit the generalizability of the results. Other platforms like Samsung Galaxy Store, Amazon Appstore, and Microsoft Store were not included, potentially missing valuable apps like “Mobile Wound Analyzer” (MOWA), which may only be accessible elsewhere. However, the App Store and Google Play remain the most popular platforms. Future research could employ web crawlers to gather a comprehensive view of available applications. Furthermore, structural differences between the App Store and Google Play have also made it impossible to compare certain aspects, such as the number of downloads per application and star ratings, which are not consistently indicated in the App Store.

The exclusion of non-French apps further affects generalizability. While the main reasons for exclusions are indicated in the table, some apps may have been excluded for multiple reasons, leading to potential underrepresentation of certain exclusions (e.g., an application removed due to the requirement of an external device may still not be available in French and vice versa). The uMARS allowed us to assess the engagement, functionality, aesthetics, and the information provided by apps. However, the equal weighting of these four categories in the final quality score may lead to suboptimal comparisons when applications have different objectives. For example, in the case of the “e-burn” app, which is used in critical care, functionality should be prioritized over engagement. Consequently, the final score may not necessarily reflect the overall quality of such a specific and purpose-driven app. Therefore, it is advisable to use uMARS to compare apps with similar objectives. As noted by Baptista et al. (2017), uMARS has limitations, including the need to refine the conceptual definition of application quality by examining how perceptions differ among healthcare professionals, researchers, and developers. The raters in this study were French-speaking Canadian nurses specialized in wound care, ostomy, and continence. Given that healthcare professionals prioritize the clinical effectiveness of mHealth apps, further research is needed to understand how experts’ perspectives influence app quality evaluation in terms of expectations, needs, preferences, and attitudes (Baptista et al., 2017).

Additionally, we conducted a descriptive evaluation of the privacy and security measures of the mobile apps. Our assessment was based on data obtained directly from the mobile apps and their descriptions in the online app stores. However, while uMARS evaluates many crucial aspects of mHealth applications, it does not specifically assess data confidentiality and security. Therefore, we recommend that future studies perform an in-depth analysis using validated tools, such as Enlight (Baumel et al., 2017).

## Conclusion

This systematic search revealed that several mobile apps for wound care are available to healthcare providers in Canada through common online app stores. However, the number of apps available in French is very limited and are all from France. As a result, recommending a single application to cover the entire clinical process—including wound assessment, prevention, and care planning—is currently challenging. This study's results indicate that the overall quality of apps available in app stores is acceptable to good. Nevertheless, considering the potential positive impact of mHealth applications in wound care and the fact that French is the second language in Canada, the Canadian stakeholders need to develop an app available in French language that is relevant in Canadian healthcare context. This involves incorporating guidelines and practices specific to the Canadian practice, including the availability of pharmaceuticals and dressings, as well as ensuring compatibility with Canadian electronic health record systems.

Nurse involvement is crucial in the development and evaluation of mHealth applications regarding quality, security, usability, and effectiveness. Furthermore, as suggested by Grundy (2022), future research should extend beyond this to encompass the broader ecosystem of mHealth. This includes considering corporate influences, national and provincial regulations, institutional policies, and the contexts in which various stakeholders must collaborate synergistically to improve wound care for those affected.

## Supplemental Material

sj-docx-1-cjn-10.1177_08445621241312394 - Supplemental material for Systematic Search and Evaluation of mobile Apps for Wound Care Available in French-Language in Canada Supplemental material, sj-docx-1-cjn-10.1177_08445621241312394 for Systematic Search and Evaluation of mobile Apps for Wound Care Available in French-Language in Canada by Julie Gagnon, Julie Chartrand, Sebastian Probst, Éric Maillet, Emily Reynolds, Valérie Chaplain, Heidi St-Jean, Raphaelle East and Michelle Lalonde in Canadian Journal of Nursing Research

sj-xlsx-2-cjn-10.1177_08445621241312394 - Supplemental material for Systematic Search and Evaluation of mobile Apps for Wound Care Available in French-Language in Canada Supplemental material, sj-xlsx-2-cjn-10.1177_08445621241312394 for Systematic Search and Evaluation of mobile Apps for Wound Care Available in French-Language in Canada by Julie Gagnon, Julie Chartrand, Sebastian Probst, Éric Maillet, Emily Reynolds, Valérie Chaplain, Heidi St-Jean, Raphaelle East and Michelle Lalonde in Canadian Journal of Nursing Research

## Notes

1. “Complex continuing care, primarily in Ontario, provides hospital-based, medically complex, and specialized services, sometimes over extended periods, that are unavailable at home or in long-term care facilities.” (CIHI, 2013, p. 5)
