# Supplementary material for: Systematic Search and Evaluation of mobile Apps for Wound Care Available in French-Language in Canada
Source: Can J Nurs Res. 2025 Feb 17;57(2):284–97. doi: 10.1177/08445621241312394 (PMC12086285; doi:10.1177/08445621241312394)
Supplement: sj-docx-1-cjn-10.1177_08445621241312394 - Supplemental material for Systematic Search and Evaluation of mobile Apps for Wound Care Available in French-Language in Canada [file sj-docx-1-cjn-10.1177_08445621241312394.docx]

Supplementary file 1

Search strategy for MEDLINE (via Ovid) database.

| Search | Query |
| --- | --- |
| 1 | Wound Healing/ |
| 2 | (wound* adj3 (car* or heal* or manag* or treat* or assess* or dress* or monitor*)) |
| 3 | (pressure adj3 (sore* or injur*) or “bedsore*”) |
| 4 | ulcer* |
| 5 | "diabetic foot" |
| 6 | dressing* |
| 7 | or/1-6 |
| 8 | Mobile Applications/ |
| 9 | Cell Phone/ |
| 10 | wireless technology/ |
| 11 | telemedicine/ |
| 12 | ("mhealth*" or "m-health*" or "m health*") |
| 13 | ((mobile or cell or phone* or portable or digital or software) adj3 app*) |
| 14 | smart tech* |
| 15 | (cellphone* or cell-phone* or cell phone* or cellular*) |
| 16 | (smartphone* or Smart phone* or smart-phone) |
| 17 | (wireless adj3 (technolog* or phone* or telephone* or device*)) |
| 18 | Tablet* |
| 19 | (hand held device* or hand-held device*) |
| 20 | (mobile adj3 (phone* or telephone* or device* or technolog*)) |
| 21 | (mobile adj3 (health* or care*)) |
| 22 | ("personal digital assistant*" or "PDA") |
| 23 | (iphone or i-phone) |
| 24 | (telemed* or tele-med*) |
| 25 | (telecar* or tele-car*) |
| 26 | (teleconsult* or tele-consult*) |
| 27 | (teledermatology or tele-dermatology) |
| 28 | (telediagnos* or tele-diagnos*) |
| 29 | (telemonitor* or tele-monitor*) |
| 30 | or/8-29 |
| 31 | 7 and 30 |
